# RETRACTION: The Impact of Long‐Term Antihypertensive Treatment on Wound Healing After Major Non‐Cardiac Surgery in Patients With Cardiovascular Diseases: A Meta‐Analysis

**DOI:** 10.1111/iwj.70588

**Published:** 2025-04-18

## Abstract

**RETRACTION:** LiuY. , MaC. , TangX. , LiuS. , and JinY. , “The Impact of Long‐Term Antihypertensive Treatment on Wound Healing After Major Non‐Cardiac Surgery in Patients With Cardiovascular Diseases: A Meta‐Analysis,” International Wound Journal 21, no. 4 (2024): e14858, 10.1111/iwj.14858.38546006 PMC10976420

The above article, published online on 28 March 2024, in Wiley Online Library (http://onlinelibrary.wiley.com/), has been retracted by agreement between the journal Editor in Chief, Professor Keith Harding; and John Wiley & Sons Ltd. Following an investigation by the publisher, all parties have concluded that this article was accepted solely on the basis of a compromised peer review process. The editors have therefore decided to retract the article. The authors did not respond to our notice regarding the retraction.

**RETRACTION:** Y. Liu , C. Ma , X. Tang , S. Liu , and Y. Jin , “The Impact of Long‐Term Antihypertensive Treatment on Wound Healing After Major Non‐Cardiac Surgery in Patients With Cardiovascular Diseases: A Meta‐Analysis,” International Wound Journal 21, no. 4 (2024): e14858, 10.1111/iwj.14858.38546006 PMC10976420

The above article, published online on 28 March 2024, in Wiley Online Library (http://onlinelibrary.wiley.com/), has been retracted by agreement between the journal Editor in Chief, Professor Keith Harding; and John Wiley & Sons Ltd. Following an investigation by the publisher, all parties have concluded that this article was accepted solely on the basis of a compromised peer review process. The editors have therefore decided to retract the article. The authors did not respond to our notice regarding the retraction.

